# Identifying Predictors and Prevalence of Alcohol Consumption among University Students: Nine Years of Follow-Up

**DOI:** 10.1371/journal.pone.0165514

**Published:** 2016-11-03

**Authors:** Lucía Moure-Rodríguez, María Piñeiro, Montserrat Corral Varela, Socorro Rodríguez-Holguín, Fernando Cadaveira, Francisco Caamaño-Isorna

**Affiliations:** 1 CIBER de Epidemiología y Salud Pública (CIBERESP), Department of Preventive Medicine, Universidade de Santiago de Compostela, Santiago de Compostela, Spain; 2 Department of Clinical Psychology and Psychobiology, Universidade de Santiago de Compostela, Santiago de Compostela, Spain; Leibniz Institute for Prvention Research and Epidemiology BIPS, GERMANY

## Abstract

**Aim:**

To evaluate the prevalence of alcohol consumption among university students during late adolescence and young adulthood and to identify the associated factors.

**Material and Methods:**

Cohort study among university students in Spain (n = 1382). Heavy Episodic Drinking (HED) and Risky Consumption (RC) were measured with the Alcohol Use Disorders Identification Test (AUDIT) at ages 18, 20, 22, 24 and 27 years. Data on potential factors associated with alcohol use were obtained with an additional questionnaire. Multilevel logistic regression for repeated measures was used to obtain adjusted OR (Odds Ratios).

**Results:**

The rates of prevalence of RC were lower, but not statistically significant, in women. The age-related changes in these rates were similar in both genders, and the prevalence of RC peaked at 20 years. By contrast, the prevalence of HED was significantly lower in women and peaked at 18 years in women and at 22 years in men. Multivariate models showed that early age of onset of alcohol use (OR = 10.6 and OR = 6.9 for women; OR = 8.3 and OR = 8.2 for men) and positive expectations about alcohol (OR = 7.8 and OR = 4.5 for women; OR = 3.6 and OR = 3.3 for men) were the most important risk factors for RC and HED. Living away from the family home was also a risk factor for both consumption patterns among women (OR = 3.16 and OR = 2.34), while a high maternal education level was a risk factor for RC among both genders (OR = 1.62 for women; OR = 2.49 for men).

**Conclusions:**

Alcohol consumption decreases significantly at the end of youth, with higher rates of prevalence and a later peak among men. Prevention strategies should focus on beliefs and expectations about alcohol and on delaying the age of onset. Women are at particular risk for these consumption patterns if they live away from their parents. Belonging to a high-income family is a strong risk factor for RC.

## Introduction

Alcohol consumption constitutes a public health problem worldwide, and Europe has the highest rate of consumption per capita [1]. In Spain, alcohol consumption is deeply rooted in social customs and until recently was traditionally associated with family and social events, with mainly men partaking [2]. However, in the last decade, heavy episodic drinking (HED), characterized by the intake of large amounts of alcohol in a short period of time (producing blood alcohol levels of at least 0.8 g/l), has replaced the traditional pattern of consumption among young people [2].

Although in the general population alcohol consumption is more prevalent in men than in women, this distinction is becoming blurred in young adults, particularly regarding HED, the prevalence of which is similar in both sexes [3]. To date, the age-related distribution of prevalence rates has followed a bell-shaped curve, peaking at around 21 years in the US [4] and at between 19 and 24 years depending on the country and gender in European studies, usually with earlier peaks in females [3,5]. One of the latest and most widely cited reviews on the topic [6] has mainly cited the following variables for HED in university students: gender, age, ethnicity, religion, expectations about alcohol, age of onset of alcohol consumption, consumption of other drugs, health and stress, personality, physical activity, socioeconomic level, living circumstances, and any family history of alcoholism. Prevalence rates of HED found in these studies can be explained by the importance of different risk factors in different European countries.

The brains of adolescents and young adults are particularly susceptible to the neurotoxic effects of HED [7–10]. Cohort studies have shown an association between this pattern of alcohol consumption and cognitive [7], structural [8] and neurofunctional effects [9]. Moreover, in the university environment, HED has been associated with poorer academic performance [11], greater consumption of medicines [12], higher incidence of injuries [13] and a higher incidence of risky sexual behavior [10], relative to control subjects. Finally, it is important to take into account that codes of behavior acquired during adolescence tend to be maintained in adulthood [14].

The objective of this study was to evaluate the prevalence of alcohol consumption among university students during adolescence and young adulthood and to identify the associated factors.

## Materials and Methods

### Design, Population and Sample

We carried out a cohort study among university students (Compostela Cohort, Spain), between November 2005 and February 2015. We used cluster sampling to select the participants. Thus, at least one of the first-year classes was randomly selected from each of the 33 university faculties/schools (a total of 53 classes). The number of classes selected in each university faculty or school was proportional to the number of students. All students present in the class on the day of the survey were invited to participate in the study (n = 1382). Abstinent students were excluded from the association analysis, although the numbers are included in the sample description. This study was approved by the Bioethics Committee of the Universidade de Santiago de Compostela. Subjects were informed both verbally and in written format, as part of the questionnaire, that participation was voluntary, anonymous, and the possibility to opt-out was available at any time. Subjects were informed that they were free to fill or refuse to fill the questionnaire. This procedure was approved by the Bioethics Committee.

### Data Collection Procedure

Two team researchers visited each first-year classroom in November 2005 and invited all students present in the class to participate in the study. Participants were evaluated via a self-administered questionnaire in the same classroom (1st questionnaire). In November 2007, the same team of researchers visited the third-year classroom in order to follow-up with the students. Participants were re-evaluated via a self-administered questionnaire (2nd questionnaire). The questionnaires were linked using birth date, sex, school, and class. Students who provided a phone number in the first or second questionnaire were further evaluated by phone at 4.5-, 6.5-, and 9.0- year follow-ups (3rd, 4rd and 5rd questionnaire). On all five occasions, alcohol use was measured with the Galician validated version of the AUDIT [15,16]. In addition to the AUDIT, we used another questionnaire that asked about the potential factors associated with alcohol use (educational level and alcohol use of parents, alcohol-related problems and age of onset of use). One of the items in the second questionnaire specifically referred to expectations about alcohol use. In this question the students were required to rank 7 positive and 7 negative expectations about the effects of alcohol. This question was generated using items from a questionnaire previously used with young Spanish adults [17]. More details about data collection are available in the following references [11,12,18].

### Definition of variables

#### Independent variables

Several socio-demographic variables were considered: gender, place of residence (parental home/away from the parental home), and maternal educational level (primary school/high school/university).

Alcohol use in the family was included as mother’s alcohol use (doesn’t consume/consumes). Four categories were defined for age of onset of use (after 16 years old, at 16, at 15, before the age of 15).

Cannabis consumption at the beginning of the study was measured with the question “Do you consume cannabis when you go out? Never; Sometimes; Most of the times; Always”. The categories were recategorized into No ("never") and Yes ("sometimes" or “most of the time” or “always”). Tobacco consumption at the beginning of the study was also measured as a dichotomous variable, without any temporal reference: No/Yes.

Finally, taking the number of positive and negative expectations into account, a score ranging from 0 to 14 was generated (0 being the maximum of negative expectancies and 14 the maximum of positive expectancies). The scores were divided into tertiles.

#### Dependent variables

1. Risky consumption (RC). Dichotomous variable generated from AUDIT score. A different cut-off value was established according to gender: = >5 for women; and = >6 for men. These cut-offs are recommended in the Galician validated version of the AUDIT.
2. Heavy episodic drinking (HED). This is a dichotomous variable generated from the third AUDIT question “How often do you have 6 or more alcoholic drinks per occasion?”, which was coded as follows: never = 0, less than once a month = 0, once a month = 1, once a week = 1, daily or almost daily = 1. The sensitivity and specificity of this question with this cut-off value are respectively 0.72 and 0.73, and the area under the curve is 0.767 (95% CI: 0.718–0.816) [19].

### Statistical analysis

We used multilevel logistic regression for repeated measures to obtain adjusted Odds Ratios (OR) for independent variables from the final RC and HED models. Confidence intervals of 95% (95% CI) were calculated. These models are more flexible than traditional models and therefore allow us to work with correlated data. As this is the case, there is a dependency structure. The same subject was measured several times, and the responses were strongly correlated. The university faculty/school and classroom were considered to be random variables. The follow-up time was included as an offset term.

Maximal models were generated, including all theoretical independent variables. From these maximal models, final models were generated. Final models included all significant variables or non-significant variables when their exclusion changed the OR of other variables by more than 10%. Data were analyzed using Generalized Linear Mixed Models in R statistical software.

## Results

The characteristics of the sample of women and men are summarised in Tables 1 and 2 respectively. There were no significant differences between the sexes in relation to any of these variables.

**Table 1:** Characteristics of female initial sample and follow-up samples.

	Percentage or mean (95%CI)					
	Initial (18–19 years old) n = 992	2-year follow-up (20–21 years old) n = 669	4-year follow-up (22–23 years old) n = 461	6-year follow-up (24–25 years old) n = 266	9-year follow-up (27–28 years old) n = 325	p-value
**Maternal educational level**						
Primary school	41.8 (38.4–45.3)	44.2 (40.1–48.4)	43.1 (38.3–48.3)	47.3 (41.3–54.1)	45.7 (40.1–51.8)	
High school	33.6 (30.2–37.1)	30.5 (26.4–34.7)	30.6 (25.8–35.8)	26.5 (20.4–33.3)	28.1 (22.5–34.2)	
University	24.6 (21.2–28.1)	25.3 (21.3–29.6)	26.3 (21.4–31.4)	26.1 (20.1–32.9)	26.2 (20.7–32.4)	0.642
**Residence**						
In parental home	24.7 (22.1–27.5)	22.9 (19.7–26.1)	22.2 (18.5–26.0)	22.1 (18.1–26.1)	20.9 (16.5–25.1)	
Away from the parental home	75.3 (72.6–78.0)	77.1 (74.0–80.3)	77.8 (74.1–81.6)	77.9 (73.9–81.9)	79.1 (74.9–83.5)	0.720
**University entrance grade**^a^						
9–10 points	45.6 (42.3–49.1)	43.2 (39.3–47.5)	43.2 (38.4–48.3)	43.3 (38–3–48.8)	47.1 (41.3–52.9)	
7 - <9 points	48.8 (45.5–52.3)	50.3 (46.4–54.6)	50.6 (45.8–55.6)	50.1 (45.1–55.6)	47.1 (41.3–52.9)	
5 - <7 points	5.6 (2.2–9.0)	6.5 (2.5–10.7)	6.2 (1.4–11.2)	6.6 (1.6–12.1)	5.8 (0–11.6)	0.977
**Positive expectations about alcohol**						
Low	37.1 (33.4–40.9)	37.5 (33.2–42.1)	36.5 (31.4–42.0)	36.5 (30.9–42.3)	37.9 (31.7–44.3)	
Medium	34.0 (30.3–37.8)	32.6 (28.3–37.3)	34.6 (29.4–40.1)	35.4 (29.8–41.1)	34.8 (28.6–41.2)	
High	28.9 (25.2–32.7)	29.9 (25.5–34.5)	28.9(23.7–34.4)	28.1 (22.5–33.8)	27.2 (21.0–33.6)	0.999
**Age of onset of use of alcohol**						
After 16 years old	19.0 (16.5–21.8)	17.9 (14.9–21.3)	16.5 (13.0–20.5)	16.7 (12.1–22.5)	14.5 (10.5–19.2)	
At 16 years old	38.9 (35.6–42.2)	38.1 (34.1–42.2)	36.8 (32.0–41.7)	40.1(33.6–46.8)	36.6 (30.9–42.6)	
At 15 years old	25.6 (22.7–28.7)	25.9 (22.3–29.6)	26.5 (22.2–31.1)	26.4 (20.8–32.7)	28.3 (23.0–34.0)	
Before age of 15 years	16.5 (14.0–19.7)	18.1 (15.0–21.5)	20.3 (16.4–24.5)	16.7 (12.1–22.5)	20.7 (16.0–25.9)	0.438
**Heavy episodic drinking**^b^						
Never	61.2 (58.2–64.3)	61.3 (57.7–65.1)	59.0 (54.7–63.7)	59.4 (53.8–65.5)	60.0 (54.8–65.4)	
Less than once a month	20.9 (17.8–23.9)	20.9 (17.3–24.7)	23.4 (19.1–28.1)	22.2 (16.5–28.3)	22.5 (17.2–27.9)	
Monthly	9.8 (6.7–12.8)	9.1 (5.5–12.9)	9.1 (4.8–13.8)	9.8 (4.1–15.9)	9.8 (4.6–15.3)	
More frequently	8.2 (5.1–11.2)	8.7 (5.1–12.5)	8.5 (4.1–13.2)	8.6 (3.0–14.8)	7.7 (2.5–13.1)	0.999
**AUDIT: Total (mean)**	5.4 (5.2–5.7)	5.6 (5.1–5.8)	5.6 (5.2–6.0)	5.6 (5.0–6.1)	5.3 (4.9–5.8)	0.884
**Cannabis consumption**	18.6 (16.2–21.1)	19.0 (15.9–22.0)	20.6 (16.8–24.4)	18.0 (13.2–22.9)	18.8 (14.4–23.2)	0.942
**Tobacco consumption**	31.0 (28.1–34.0)	31.5 (27.9–35.1)	34.3 (29.8–38.7)	31.2 (25.4–37.0)	32.9 (27.7–38.2)	0.786

^a^ Variable with a scale of 1 to 10

^b^ Question 3 of the AUDIT.

**Table 2:** Characteristics of male initial sample and follow-up samples.

	Percentage or mean (95%CI)					
	Initial (18–19 years old) n = 371	2-year follow-up (20–21 years old) n = 206	4-year follow-up (22–23 years old) n = 139	6-year follow-up (24–25 years old) n = 81	9-year follow-up (27–28 years old) n = 90	p-value
**Maternal educational level**						
Primary school	32.0 (26.5–37.8)	35.8 (28.4–43.3)	41.6 (32.8–50.8)	43.0 (31.6–54.8)	41.6 (31.5–53.5)	
High school	27.6 (22.1–33.3)	27.4 (19.9–34.9)	25.5 (16.8–34.7)	24.1 (12.7–35.8)	27.0 (16.8–38.9)	
University	40.3 (34.8–46.0)	36.8 (29.3–44.3)	32.8 (24.1–42.0)	32.9 (21.5–44.7)	31.5 (21.3–43.4)	0.449
**Residence**						
In the parental home	29.7(25.1–34.5)	27.8 (21.9–34.1)	28.8 (21.6–36.4)	31.6 (23.9–40.6)	28.9 (20.0–38.3)	
Away from the parental home	70.3 (65.7–75.1)	72.2 (66.3–78.5)	71.2 (64.0–78.9)	68.4 (60.7–77.4)	71.7 (62.2–80.5)	0.949
**University entrance grade**^a^						
9–10 points	50.7 (45.3–56.2)	47.5 (40.6–55.0)	50.0 (42.0–59.2)	50.0 (41.4–60.1)	51.7 (41.6–62.7)	
7 - <9 points	42.7 (37.3–48.2)	43.6 (36.6–51.0)	43.5 (35.5–52.7)	42.2 (33.6–52.3)	42.7 (32.6–53.7)	
5 - <7 points	6.6 (1.1–12.1)	8.9 (2.0–16.4)	6.5 (0–15.7)	7.8 (0–17.8)	5.6 (0–16.6)	0.996
**Positive expectations about alcohol**						
Low	29.7 (23.7–36.0)	33.0 (25.1–41.0)	34.2 (25.0–44.3)	35.4 (25.3–46.4)	31.6 (20.3–43.7)	
Medium	38.0 (32.0–44.4)	30.7 (22.9–38.8)	31.7 (22.5–41.8)	32.3 (22.2–43.4)	30.4 (19.0–42.5)	
High	32.3 (26.3–38.7)	36.3 (28.5–44.4)	34.2 (25.0–44.3)	32.3 (22.2–43.4)	38.0 (26.6–50.0)	0.705
**Age of onset of alcohol use**						
After 16 years old	18.1 (12.5–24.1)	16.8 (9.2–24.7)	15.5 (6.9–25.5)	16.4 (6.0–29.7)	18.2 (7.8–30.3)	
At 16 years old	36.9 (31.2–42.8)	41.0 (33.5–49.0)	44.0 (35.3–54.0)	50.7 (40.3–64.0)	48.1 (37.7–60.1)	
At 15 years old	21.6 (15.9–27.5)	20.2 (12.7–28.2)	21.6 (12.9–1.6)	23.9 (13.4–37.2)	20.8 (10.4–32.8)	
Before age of 15 years	23.4 (17.8–29.4)	22.0 (14.4–30.0)	19.0 (10.3–9.0)	9.0 (0.0–22.3)	13.0 (2.6–25.1)	0.381
**Heavy episodic drinking**^b^						
Never	39.1 (34.0–44.7)	43.2 (36.4–50.6)	42.4 (34.5–51.7)	46.9 (37.0–58.9)	45.6 (35.6–56.5)	
Less than once a month	25.3 (20.2–31.0)	20.4 (13.6–27.8)	21.6 (13.7–30.8)	21.0 (11.1–33.0)	21.1 (11.1–32.1)	
Monthly	12.7 (7.5–18.3)	14.6 (7.8–22.0)	13.7 (5.7–22.9)	17.3 (7.4–29.3)	15.6 (5.6–26.5)	
More frequently	22.9 (17.8–28.6)	21.8 (15.0–29.2)	22.3 (14.4–31.6)	14.8 (4.9–26.8)	17.8 (7.8–28.8)	0.905
**AUDIT: Total (mean)**	7.8 (7.2–8.4)	7.4 (6.6–8.2)	7.3 (6.4–8.2)	6.5 (5.4–7.6)	7.1 (6.0–8.2)	0.784
**Cannabis consumption**	27.0 (22.3–31.6)	27.7 (21.3–34.0)	25.9 (18.3–33.5)	23.5 (13.6–33.3)	24.4 (15.0–33.9)	0.885
**Tobacco consumption**	27.5 (22.8–32.2)	21.8 (16.0–27.7)	23.0 (15.7–30.4)	23.5 (13.6–33.3)	24.4 (15.0–33.9)	0.636

^a^ Variable with a scale of 1 to 10

^b^ Question 3 of the AUDIT.

**Fig 1:**
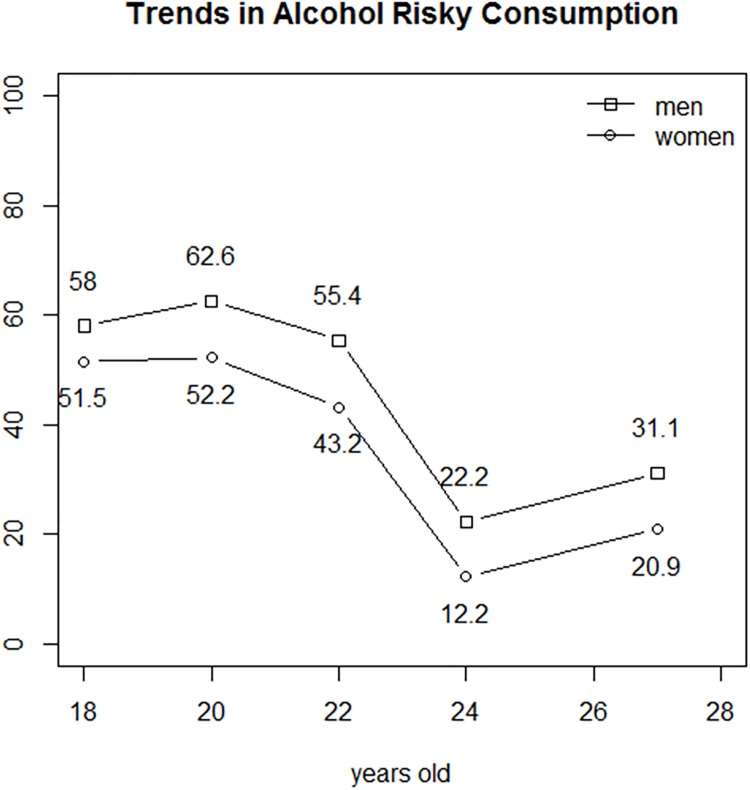
Prevalences of risky consumption among female and male Spanish university students from 18–19 years old to 27–28 years old.

**Fig 2:**
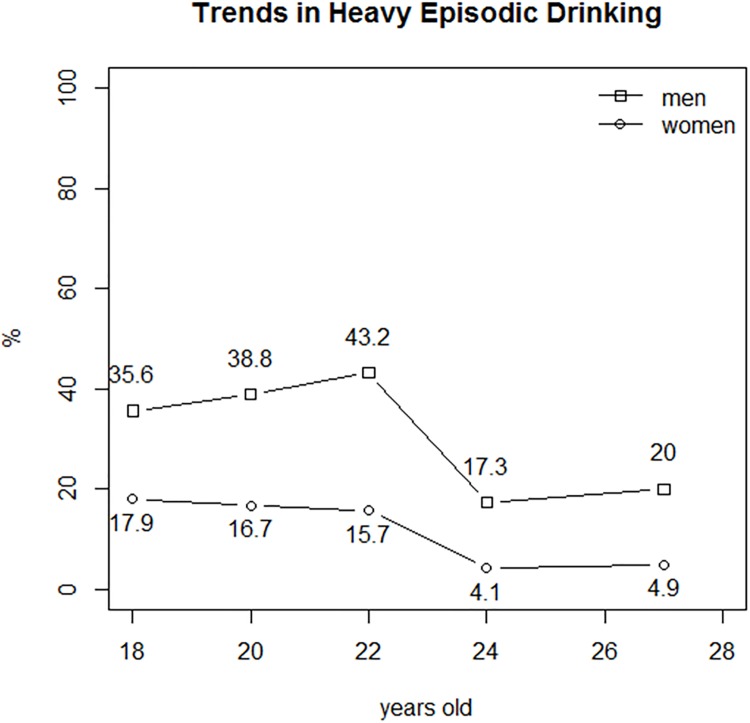
Prevalences of heavy episodic drinking among female and male spanish university students from 18–19 years old to 27–28 years old.

The rates of prevalence of RC were always lower in women, although the differences were not statistically significant. The age-related changes in these rates were similar in men and women, and the prevalence of RC peaked at 20 years. By contrast, the prevalence of HED was significantly lower in women of all ages and peaked at 18 years in women and at 22 years in men. The rates of prevalence of RC and HED in each age group of women and men are shown in Tables 3 and 4 respectively. Figs 1 and 2 present the trends of prevalences of RC and HED, for both sexes.

**Table 3:** Main characteristics of the female subjects and alcohol consumption at age 18, 20, 22, 24 and 27 years.

	Percentage of Risky Consumption					Percentage of Heavy Episodic Drinking				
	Age in years					Age in years				
	18–19	20–21	22–23	24–25	27–28	18–19	20–21	22–23	24–25	27–28
**Maternal educational level**										
Primary school	47.2	46.1	39.1	12.0	18.9^	17.1	12.6	11.2	5.6	4.7^
High school	53.5	55.9	42.9	11.4	16.5^	18.5	19.3	20.0	4.3	4.4^
University	57.3*	58.9*	51.7	13.0	29.4^	18.7	21.4*	18.3	1.4	5.9^
**Residence**										
In parental home	42.2*	40.1*	36.3	9.5	14.7^	13.9	11.2*	12.7	3.2	0*^
Away from the parental home	54.9	56.1	45.4	12.9	22.6^	19.4	18.5	16.5	4.5	6.2^
**University entrance grade**^a^										
9–10 points	40.4	41.5	29.6	0	22.2^	5.8	12.2	22.2	0	5.6
7 - <9 points	50.8	49.5	37.6	11.7	18.5^	16.2	16.9	12.2	3.1	2.1^
5 - <7 points	53.6	57.3	51.3*	12.1	22.6^	20.8*	17.9	19.6	3.7	6.8^
**Positive expectations about alcohol**										
Low	27.5	33.3	25.0	5.4	10.9^	7.1	6.9	7.4	2.2	3.6
Medium	64.3	60.6	49.3	18.8	18.8^	19.4	17.6	18.6	6.2	3.0^
High	71.0*	64.5*	59.8*	12.1*	32.9*^	27.4*	26.7*	19.7*	3.0	6.3^
**Age of onset of alcohol use**										
After 16 years old	30.5	42.3	28.8	7.9	2.5^	7.9	8.7	10.6	7.9	0
At 16 years old	57.3	57.0	44.2	14.3	22.8^	13.4	18.1	17.7	5.5	3.0^
At 15 years old	69.2	70.0	61.3	11.7	28.2^	28.5	22.7	20.8	0	7.7^
Before age 15 years	78.9*	63.8*	56.8*	23.7	33.3*^	38.7*	26.7*	21.0	7.9	10.5^
**Cannabis consumption at 18 years old**										
No	42.9	45.8	37.2	9.6	14.8^	13.3	12.0	12.6	3.7	2.7^
Yes	89.2*	79.5*	66.3*	22.9*	47.5*^	38.4*	37.0*	27.4*	6.2	14.8*^
**Tobacco consumption at 18 years old**										
No	37.7	42.4	34.0	9.8	16.1^	10.5	12.2	12.9	3.8	3.7^
Yes	82.1*	73.5*	60.8*	16.9	30.8*^	34.4*	26.5*	20.9*	4.8	7.5^
**Total**	51.5	52.2	43.2	12.2	20.9^	17.9	16.7	15.7	4.1	4.9^

^a^ Variable with a scale of 1 to 10.

*Significant differences in relation to category, p<0.05.

^ Significant differences in relation to age group, p<0.05.

**Table 4:** Main characteristics of the male subjects and alcohol consumption at ages 18, 20, 22, 24 and 27 years.

	Percentage of Risky Consumption					Percentage of Heavy Episodic Drinking				
	Age in years					Age in years				
	18–19	20–21	22–23	24–25	27–28	18–19	20–21	22–23	24–25	27–28
**Maternal educational level**										
Primary school	47.4	59.7	45.6	14,7	24.3^	29.3	36.1	31.6	8.8	18.9^
High school	60.0	58.2	65.7	10.5	29.2^	38.0	41.8	51.4	10.5	25.0^
University	65.8*	70.3	57.8	38.5*	42.9^	39.0	40.5	48.9	30.8	17.9
**Residence**										
In parental home	51.4	56.1	47.5	18.5	19.2^	34.9	41.9	30.0*	14.8	7.7^
Away from the parental home	60.5	64.9	58.6	24.1	35.9^	35.7	31.6	48.5	18.5	25.0^
**University entrance grade**^a^										
9–10 points	47.8	61.1	44.4	20.0	0	21.7	22.2	33.3	0	0
7 - <9 points	51.3	55.7	55.0	26.5	36.8^	31.3	34.1	36.7	20.6	28.9
5 - <7 points	64.6*	69.8	56.5	19.0	28.3^	41.0	45.8	50.7	16.7	13.0^
**Positive expectations about alcohol**										
Low	33.7	42.4	39.0	10.3	32.0^	20.2	25.4	19.5	3.4	20.0
Medium	64.0	70.9	55.3	36.8	41.7^	37.7	41.8	42.1	31.6	29.2
High	73.2*	75.4*	68.3*	26.1	23.3^	46.4*	46.2*	65.9*	17.4*	16.7^
**Age of onset of alcohol use**										
After 16 years old	32.8	62.1	61.1	18.2	28.6^	17.2	37.9	38.9	9.1	21.4
At 16 years old	60.2	71.8	66.7	38.2	32.4^	31.4	40.8	47.1	32.4	21.6
At 15 years old	73.9	68.6	56.0	12.5	31.2^	46.4	40.0	52.0	12.5	18.8^
Before age of 15 years	89.3*	86.8	68.2	0	50.0^	66.7*	65.8*	59.1	0	20.0^
**Cannabis consumption at 18 years old**										
No	45.4	57.3	47.6	17.7	26.5^	22.1	27.5	35.0	11.3	14.7^
Yes	92.0*	86.0*	77.8*	36.8	45.5^	72.0*	68.4*	66.7*	36.8*	36.4*^
**Tobacco consumption at 18 years old**										
No	47.6	56.5	51.4	17.7	30.9^	25.7	34.2	36.4	11.3	20.6^
Yes	85.3*	84.4*	68.8	36.8	31.8^	61.8*	55.6*	65.6*	36.8*	18.2^
**Total**	58.0	62.6	55.4	22.2	31.1^	35.6	38.8	43.2	17.3	20.0^

^a^ Variable with a scale of 1 to 10.

*Significant differences in relation to category, p<0.05.

^ Significant differences in relation to age group, p <0.05.

In relation to the factors associated with RC and HED, the multivariate model revealed the following as risk factors in both sexes: early onset of alcohol consumption (OR = 10.7 and OR = 6.90 in women compared with OR = 8.30 and OR = 2.23 in men) and positive expectations about alcohol (OR = 7.80 and OR = 4.54 in women compared with OR = 3.59 and OR = 3.26 in men). These data are presented in Tables 5 and 6.

**Table 5:** Influence of characteristics of female subjects and family background on risky consumption and heavy episodic drinking: Generalized Linear Mixed Models.

	Risky Consumption		Heavy Episodic Drinking	
	Odds Ratio (95%CI)		Odds Ratio (95%CI)	
	Bivariate	Multivariate^a^	Bivariate	Multivariate^a^
**Maternal educational level**				
Primary school	1	1		
High school	1.56 (1.10–2.20)	1.02 (0.68–1.54)		
University	1.93 (1.32–2.83)	1.62 (1.03–2.56)		
**Residence**				
In parental home	1	1	1	1
Away from the parental home	2.09 (1.45–3.03)	3.16 (2.07–4.82)	2.00 (1.18–3.38)	2.34 (1.43–3.84)
**Positive expectations about alcohol**				
Low	1	1	1	1
Medium	5.90 (3.97–8.78)	6.27 (3.96–9.92)	4,15 (2.37–7.28)	3.04 (1.81–5.12)
High	8.65 (5.68–13.17)	7.80 (4.83–12.60)	6.77 (3.79–12.07)	4.54 (2.67–7.70)
**Age of onset of alcohol use**				
After 16 years old	1	1	1	1
At 16 years old	3.16 (2.09–4.78)	4.10 (2.41–6.98)	2.15 (1.18–3.91)	2.20 (1.14–4.22)
At 15 years old	5.35 (3.43–9.35)	7.20 (4.05–12.81)	4.56 (2.42–8.56)	4.18 (2.12–8.26)
Before age of 15 years	6.44 (3.94–10.52)	10.65 (5.63–20.14)	7.07 (3.60–13.91)	6.90 (3.36–14.18)
**Age of participants**				
18–19 years	1	1	1	1
20–21 years	1,00 (0.75–1. 32)	0.84 (0.61–1.14)	0.81 (0.81–0.82)	0.87 (0.61–1.25)
22–23 years	0.48 (0.35–0.67)	0.43 (0.30–0.61)	0.76 (0.76–0.77)	0.75 (0.50–1.12)
24–25 years	0.03 (0.02–0.05)	0.03 (0.02–0.05)	0.09 (0.09–0.09)	0.14 (0.07–0.30)
27–28 years	0.08 (0.05–0.12)	0.06 (0.04–0.10)	0.10 (0.10–0.10)	0.13 (0.06–0.26)

^a^ Adjusted by all variables included in the column.

**Table 6:** Influence of characteristics of male subjects and family background on risky consumption and heavy episodic drinking: Generalized Linear Mixed Models.

	Risky Consumption		Heavy Episodic Drinking	
	Odds Ratio (95%CI)		Odds Ratio (95%CI)	
	Bivariate	Multivariate^a^	Bivariate	Multivariate^a^
**Maternal educational level**				
Primary school	1	1	1	1
High school	1.95 (1.01–3.75)	1.72 (0.72–4.09)	2.10 (1.10–4.03)	1.33 (0.62–2.85)
University	3.26 (1.75–6.08)	2.49 (1.11–5.56)	2.37 (1.28–4.36)	1.92 (0.94–3.93)
**Positive expectations about alcohol**				
Low	1	1	1	1
Medium	4.80 (2.35–9.80)	2.33 (0.97–5.62)	3.19 (1.57–6.48)	1.80 (0.82–3.94)
High	5.64 (2.73–11.65)	3.59 (1.48–8.74)	4.47 (2.17–9.22)	3.26 (1.48–7.18)
**Age of onset of alcohol use**				
After 16 years old	1	1	1	1
At 16 years old	2.93 (1.42, 6.08)	3.28 (1.22–8.83)	2.15 (1.00–4.61)	1.83 (0.75–4.45)
At 15 years old	4.19 (1.79–9.84)	3.88 (1.24–12.14)	3.61 (1.52–8.60)	3.42 (1.24–9.44)
Before age of 15 years	14.19 (5.60–35.97)	8.30 (2.49–27.60)	10.48 (4.20–26.15)	8.23 (2.80–24.22)
**Age of participants**				
18–19 years	1	1	1	1
20–21 years	1.53 (0.94–2.51)	1.83 (1.00–3.36)	1.26 (0.79–2.01)	1.35 (0.79–2.29)
22–23 years	0.85 (0.49–1.49)	0.79 (0.41–1.54)	1.74 (1.02–2.98)	1.84 (1.00–3.41)
24–25 years	0.09 (0.04–0.20)	0.07 (0.03–0.20)	0.29 (0.13–0.64)	0.29 (0.12–0.70)
27–28 years	0.15 (0.07–0.31)	0.11 (0.05–0.27)	0.33 (0.16–0.68)	0.34 (0.15–0.77)

^a^ Adjusted by all variables included in the column.

Older age of participants constituted a protective factor (OR = 0.06 and OR = 0.13 in women compared with OR = 0.11 and OR = 0.34 in men), and the effect was greater in women than in men for both patterns of consumption. Finally, in relation to the sociodemographic characteristics of the family, high maternal educational level acted as a risk factor only for RC (OR = 1.62 in women compared with OR = 2.49 in men), while the place of residence was significant only in women for both RC and HED (OR = 3.16 and OR = 2.34). These data are presented in Tables 5 and 6.

## Discussion

The findings indicate high rates of prevalence of both RC and HED in the population of university students under study. Moreover, multivariate models showed that early age of onset of drinking and positive expectations about alcohol were the most important risk factors for RC and HED respectively. Finally, living away from the family home also constituted a risk factor for both patterns consumption among women, while a high maternal educational level was found to be a risk factor for RC in both women and men.

The distribution of changes in prevalence of HED followed a bell-shaped curve, similar to that described in previous studies [4], although with an earlier peak in women than in men. Although the HED pattern is generally considered to peak during the vital life stage between adolescence and young adulthood [20,21], which has been explained as a consequence of the acquisition of new social roles and responsibilities at this stage [22], this was only confirmed in women in the present study. We found that one in five men of age 27 years continued to follow this pattern of alcohol consumption. These findings should be considered in the current social and economic situation in Spain, which is undergoing an economic crisis that is particularly affecting young adults and greatly limiting their access to the job market [23]. This may cause delay in the acquisition of new roles and responsibilities that are understood to be part of this new life stage of passage into adulthood [20]. Finally, in relation to differences between the sexes, HED was not as prevalent in females as in males. However, it is possible that the prevalence of HED in women may have been underestimated, as the 3rd question of the AUDIT considers consumption of 6 or more drinks.

This same hypothesis of new roles and responsibilities can be one explanation for the important decrease observed at age of 24 years for both consumption patterns, irrespective of group or gender. After college years, when high prevalences of consumption are observed, young people become concerned about their futures, and try to enter the job market and acquire new responsibilities. In fact, during this ages, students who entered college with better grades, declared no HED or RC practices at all. Moreover, the slight increase at age 27 may be due to new adult alcohol consumption.

Regarding the factors associated with RC and HED, early onset of alcohol consumption was found to be the main risk factor for both patterns of drinking. This is consistent with the findings of previous studies, in which early onset of drinking has been associated with higher consumption and negative consequences during adolescence and with problems related to alcohol abuse and dependency in adults [24]. For example, Cranford [25] showed that the risk of HED in university students was up to four times higher in students who had begun drinking at earlier stages (OR = 4.15), establishing this variable as an important predictor of HED independently of race or gender. Greater parental permissiveness has been indicated as an explanatory factor for this association. Previous studies have shown that alcohol consumption in adolescents is affected by the perception that the young people have of their parents’ attitude to drinking [26,27]. Children of more permissive parents begin to drink at earlier ages and undertake risky drinking behavior during adolescence and early adulthood.

In the present study, positive expectations about alcohol acted as a risk factor for RC and HED in both sexes. This is consistent with previously reported findings, in relation to both the RC pattern [28,29] and the HED pattern [30,31]. We also found differences between the sexes in relation to expectations about alcohol, as previously reported [32,33], with this factor having a greater influence in females.

Socioeconomic level clearly influences alcohol consumption in adults, although the effect is not as evident in young people, and variable results have been reported [34]. Nevertheless university students have been found to be particularly strongly affected by the price of alcoholic beverages in other studies [35]. In our study maternal educational level only influenced RC, which was more prevalent in students whose mothers had completed secondary or higher education studies. This variable is understood by many authors as a reflection of socioeconomic level [3,17]. The lack of association between maternal educational level and HED supports this hypothesis of being a reflection of the socioeconomic status. This is due to the fact that one of the main characteristics of the phenomenon of street drinking that has become popular among young people in Spain is that alcohol can be consumed at a relatively low cost [36]. Finally, maternal alcohol use did not reveal any association with any dependent variables.

Living away from the family home proved to be a risk factor for both patterns of consumption, although only in women. Some researchers consider that stricter, more controlled home environments may constitute a protective factor for alcohol consumption [37,38]. In a country where alcohol consumption has traditionally been associated with men, the home environment may be stricter as regards preventing excessive drinking in young females [39]. Aside from the quality of the relationship between young adults and their parents, parental attitudes towards alcohol consumption have been found especially influential among females [25,40]. Our findings indicate that living in one’s family home has a greater effect among women than the peer group. These findings are consistent with the previous studies [28,38–41].

There are four main limitations to this study: 1) selection bias, because of the loss of subjects at follow-up. However, the absence of significant differences between the initial samples and the follow-up samples suggest the absence of this bias; 2) self-reported data may be skewed due to inconsistent personal feelings or memories. However, the AUDIT questionnaire has been internationally validated in adolescents and young adults; 3) the most appropriate definition of HED in Spain implies differences between genders: more than five alcoholic beverages for women and more than six for men, on a single occasion. The third question of the AUDIT therefore underestimates the prevalence of HED in women. However, this limitation will mainly affect descriptive and not analytical statistics. The use of a gender-specific instrument instead of AUDIT could be advisable in future studies; and 4) given that the question about expectancies is not specifically validated, expectancies may not have been correctly measured.

In conclusion, alcohol consumption declines significantly at the end of young adulthood, with higher rates of prevalence and a later peak among men than among women. Prevention strategies should focus on expectations and beliefs about alcohol and on delaying the age of onset of alcohol use. Women are particularly at risk for these consumption patterns when living away from the family home. Being from a high-income family is a strong risk factor for risky consumption.
